# Supplementary material for: Structural basis of mRNA decay by the human exosome–ribosome supercomplex
Source: Nature. 2024 Oct 9;635(8037):237–42. doi: 10.1038/s41586-024-08015-6 (PMC11540850; doi:10.1038/s41586-024-08015-6)

nature portfolio

https://doi.org/10.1038/s41586-024-08015-6

Supplementary information

Structural basis of mRNA decay by the human exosome–ribosome supercomplex

In the format provided by the authors and unedited

Raw SDS-PAGE gel, Figure 1b

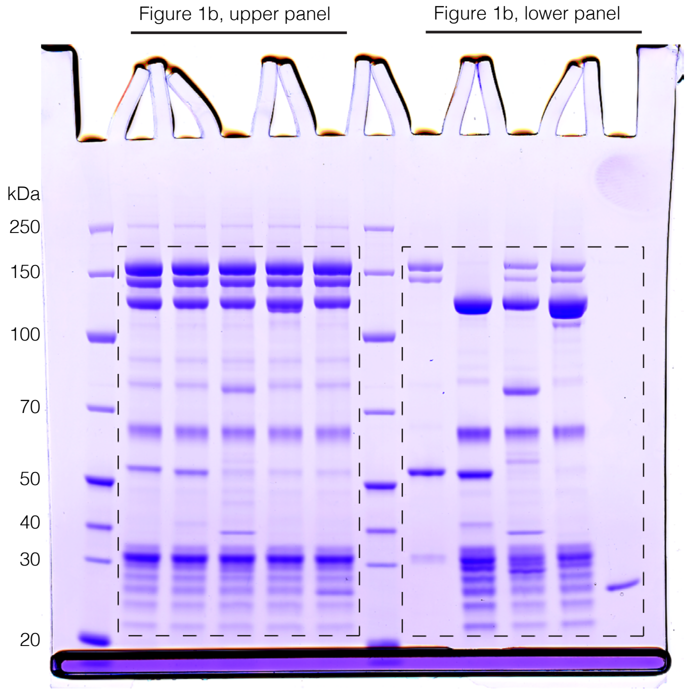

Raw Western Blot images, Figure 4b

RNCs only

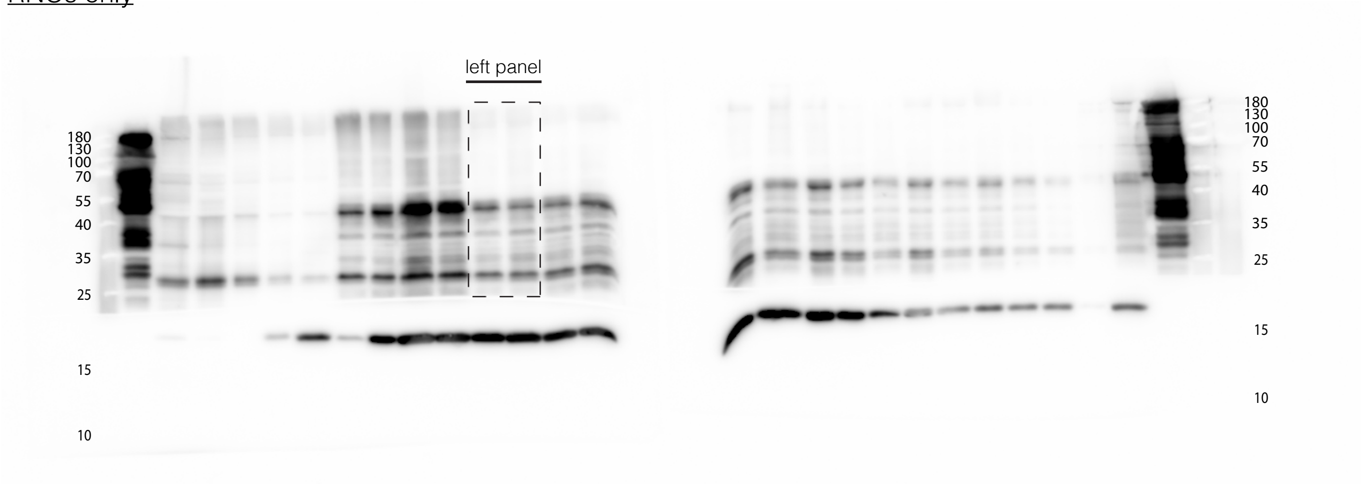

RNCs + SKI2$_{\Delta Wedge}$38

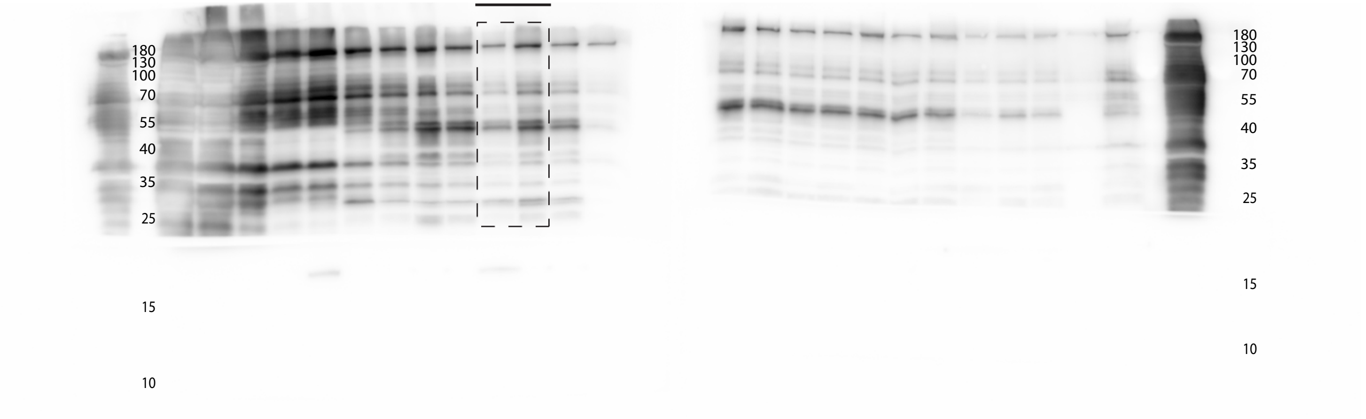

RNCs + SKI2$_{N}$38 gatekeeping module

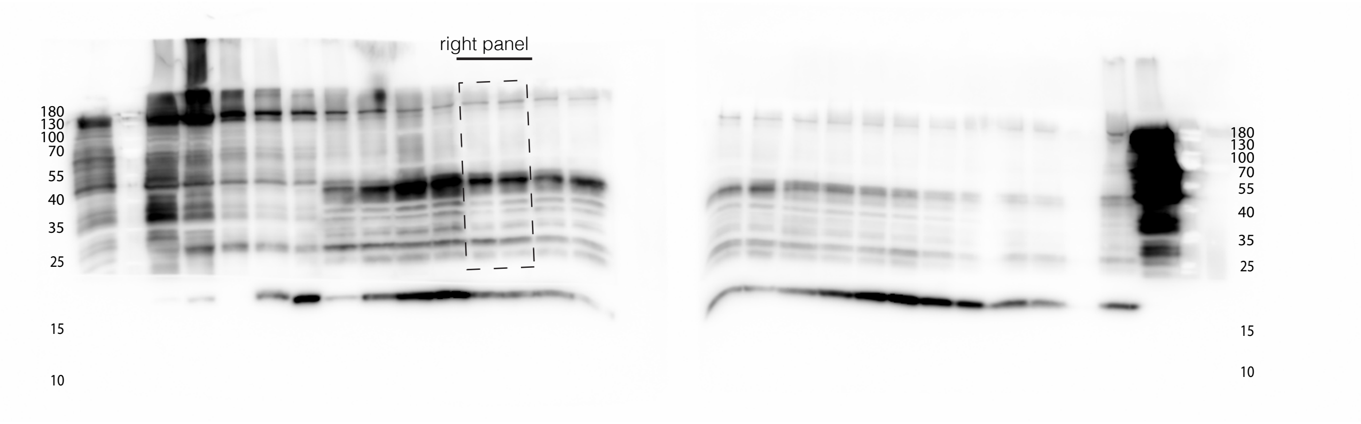

Raw SDS-PAGE gels, Extended Data Figure 1c

Extended Data Figure 1c, upper panel

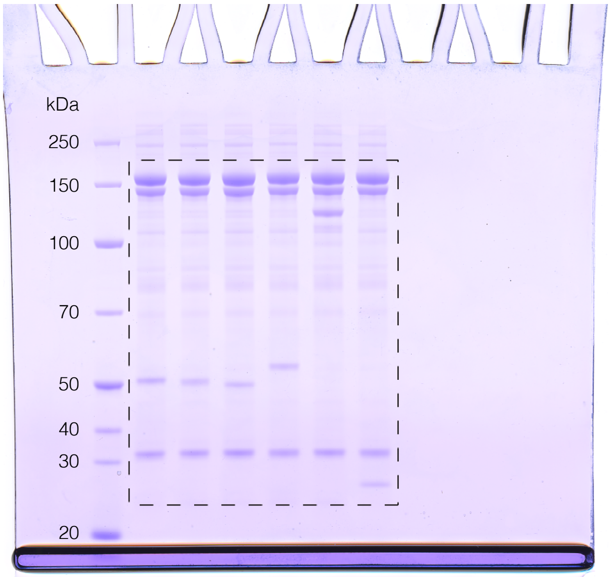

Extended Data Figure 1c, lower panel

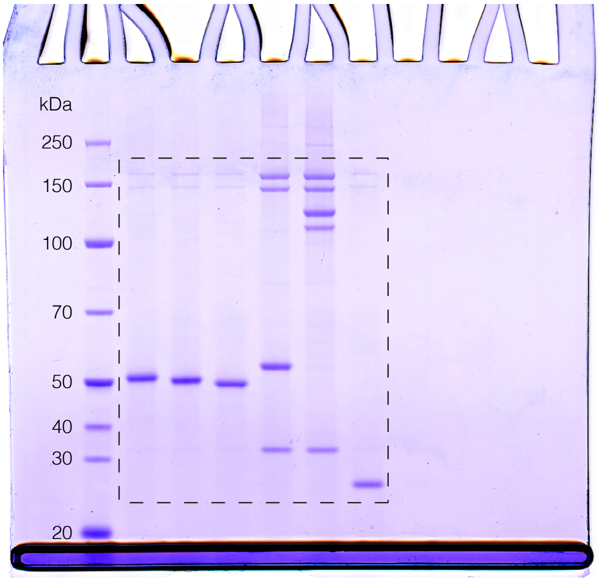

Raw SDS-PAGE gels, Extended Data Figure 1d

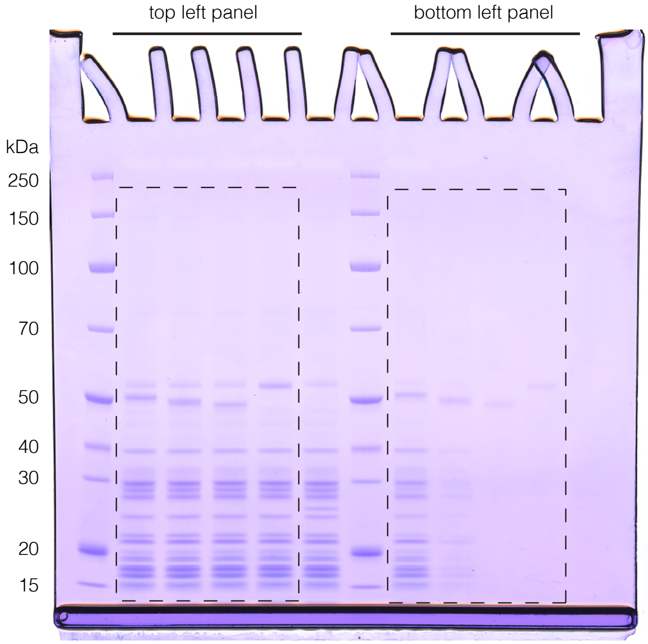

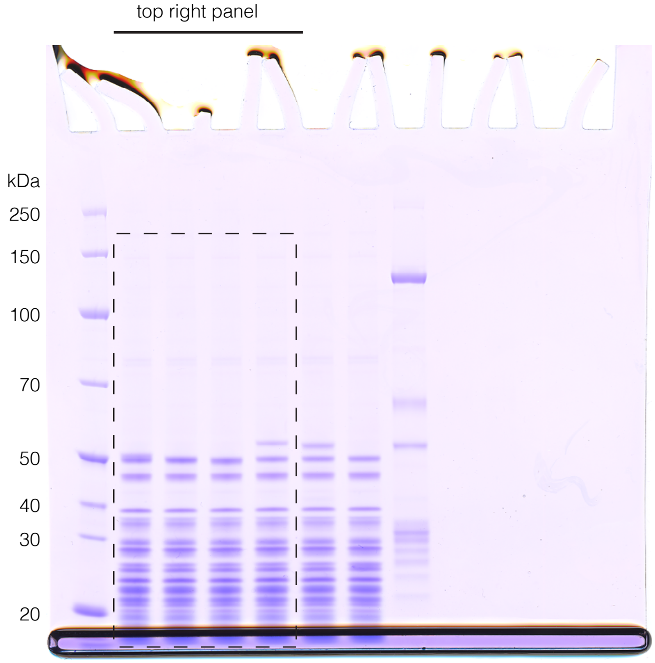

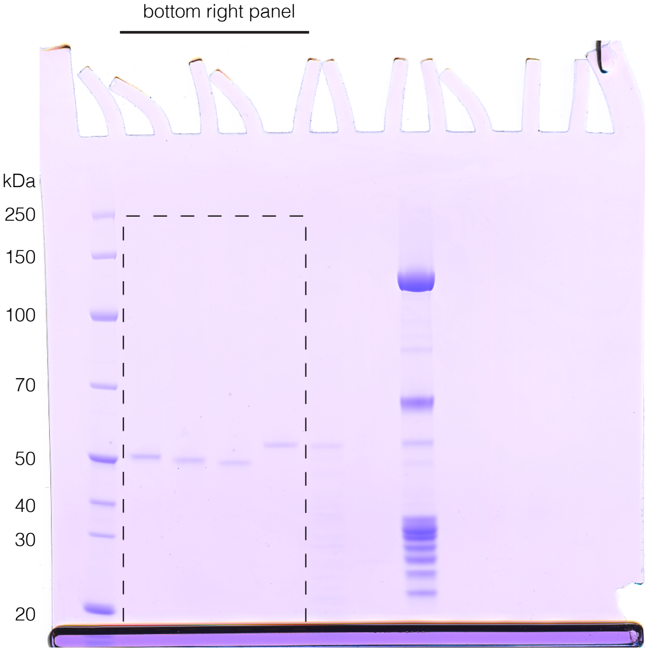

Raw UREA-PAGE gels, Extended Data Figure 1f

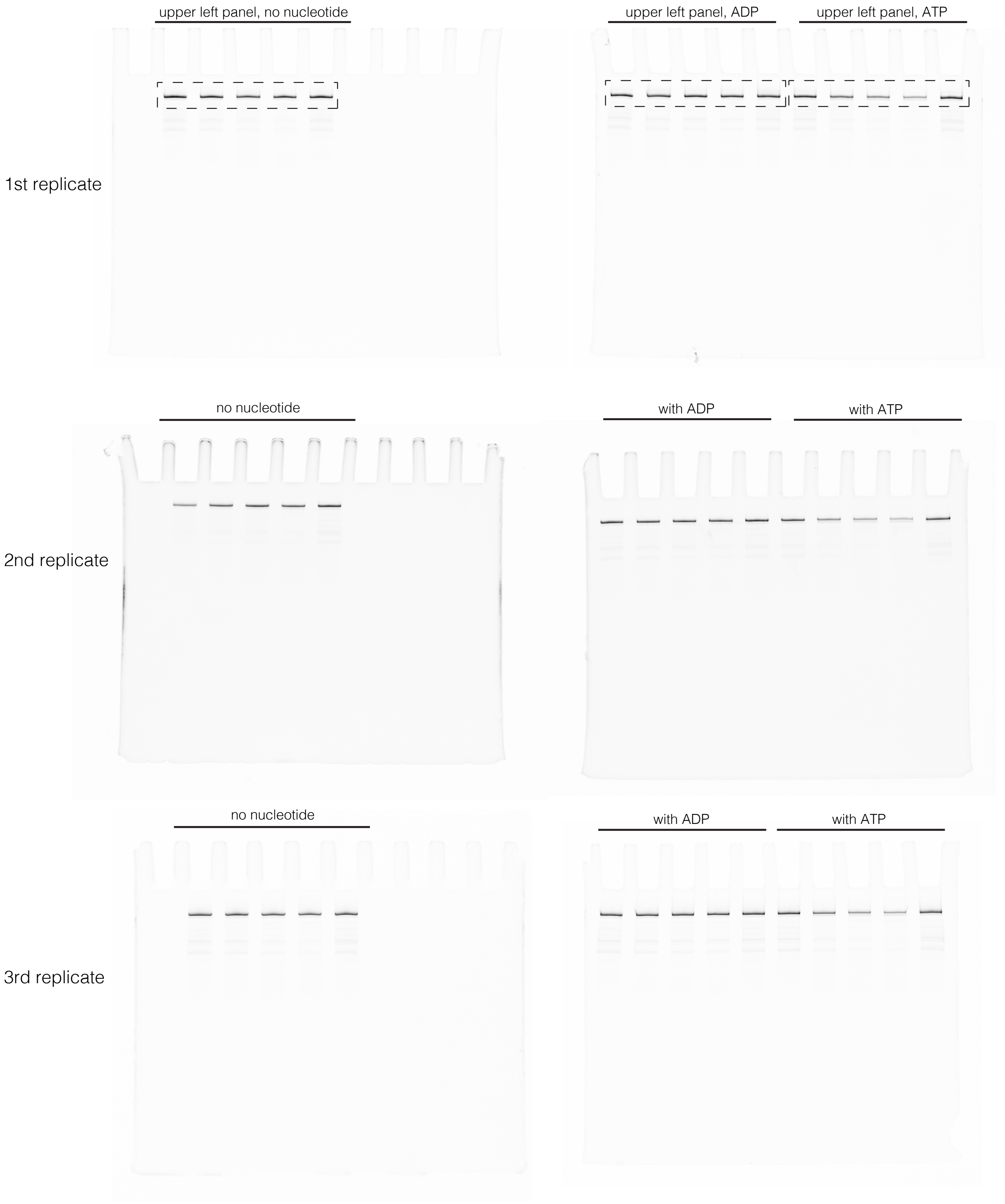

Raw SDS-PAGE gel, Extended Data Figure 1f

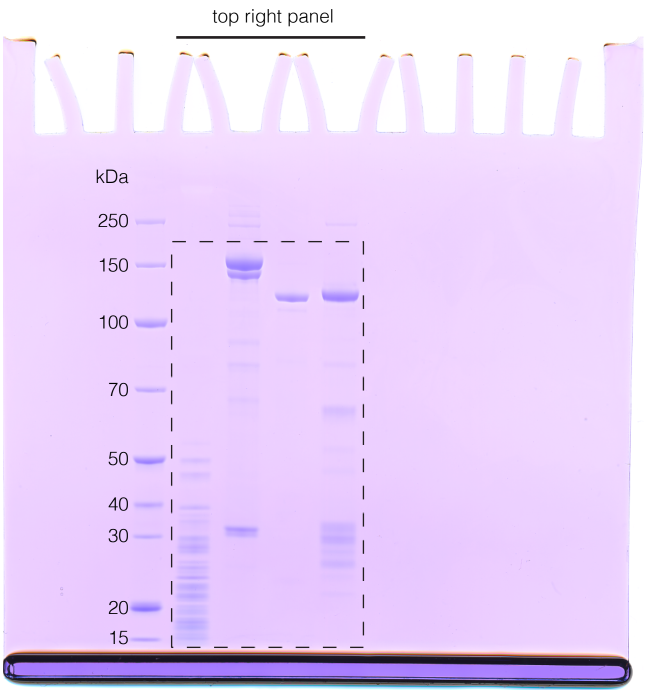

Raw SDS-PAGE gel, Extended Data Figure 2a

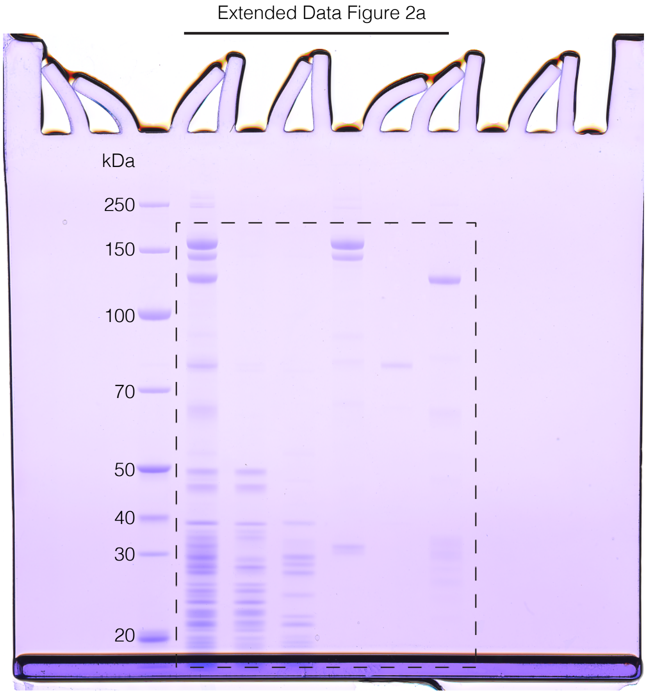

Raw SDS-PAGE gel, Extended Data Figure 4a

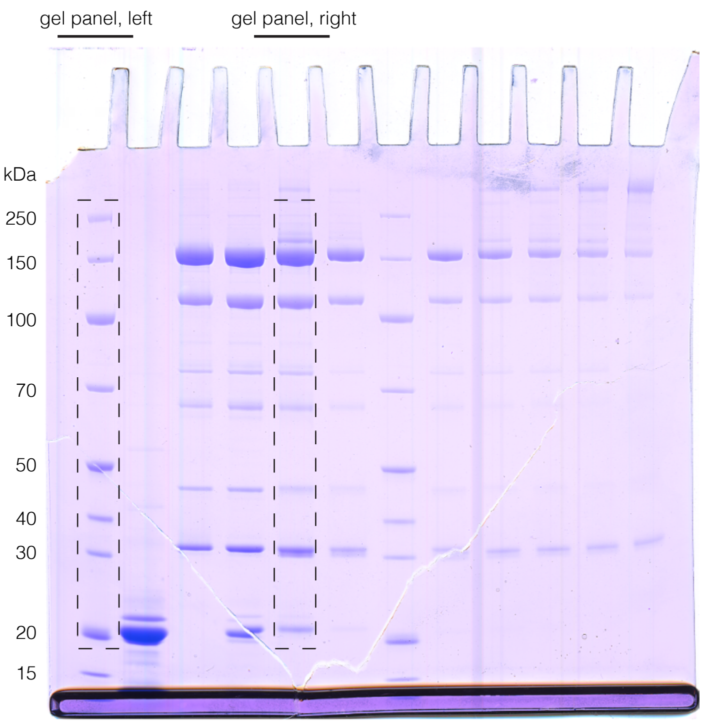

Raw SDS-PAGE gel, Extended Data Figure 4h

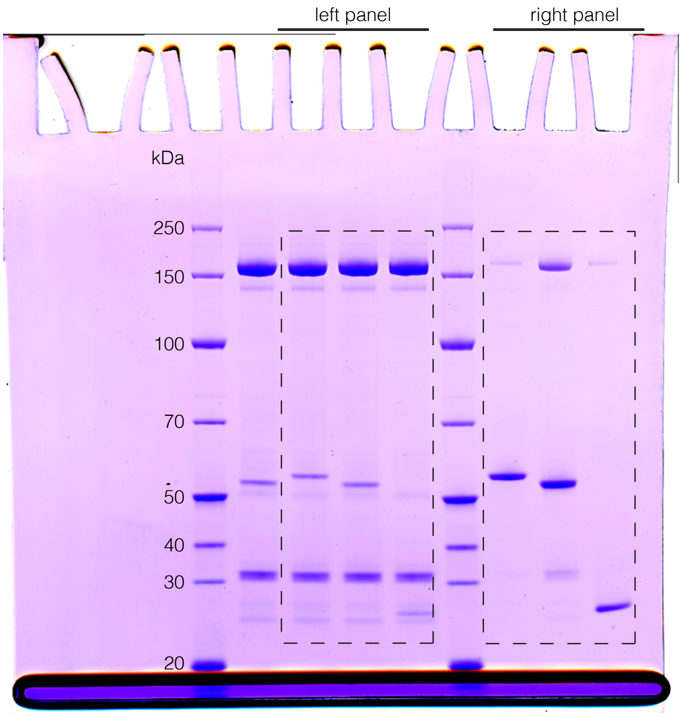

Raw Western Blot images, Extended Data Figure 9b

Panel 1: stalled RNCs

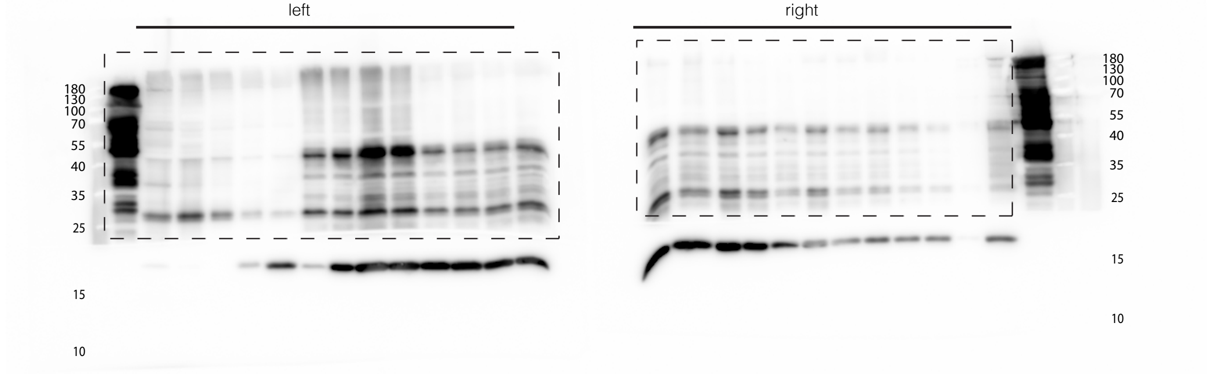

Panel 2: stalled RNCs + SKI2$_{\Delta Wedge}$38

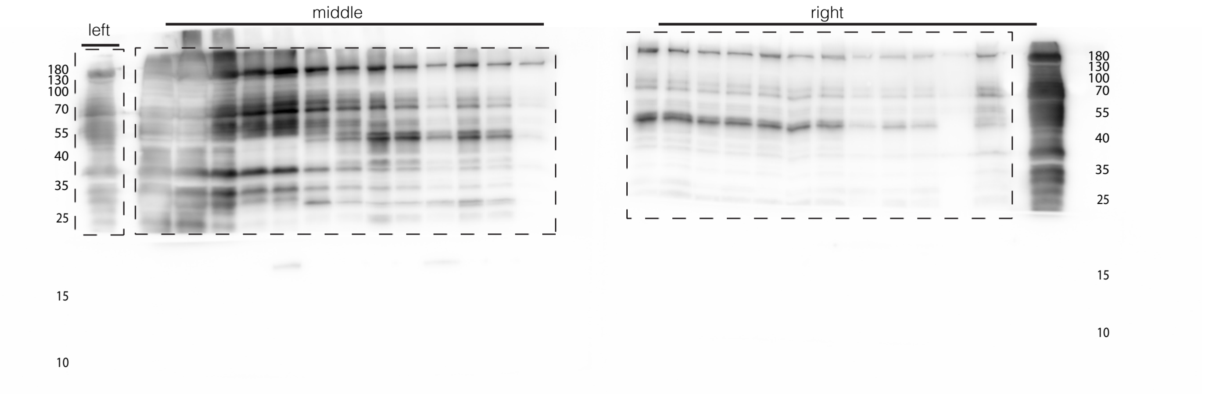

Panel 3: stalled RNCs + SKI2$_{N}$38

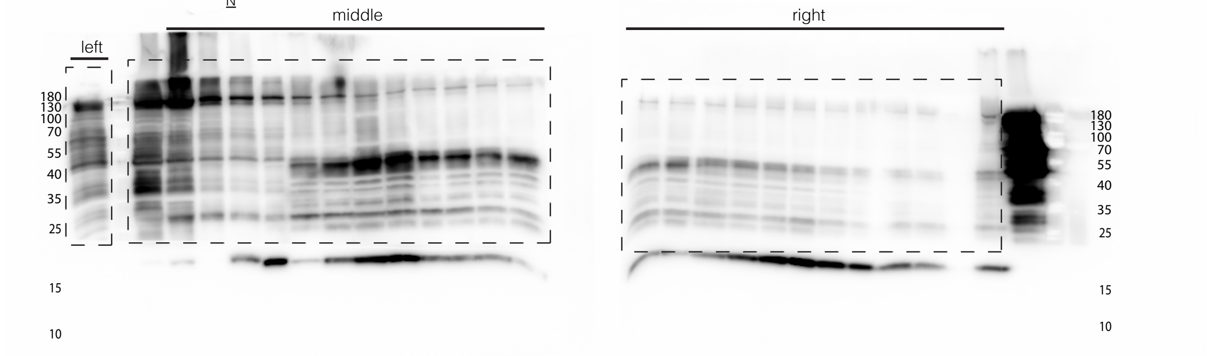

Panel 4: SKI2$_{N}$38

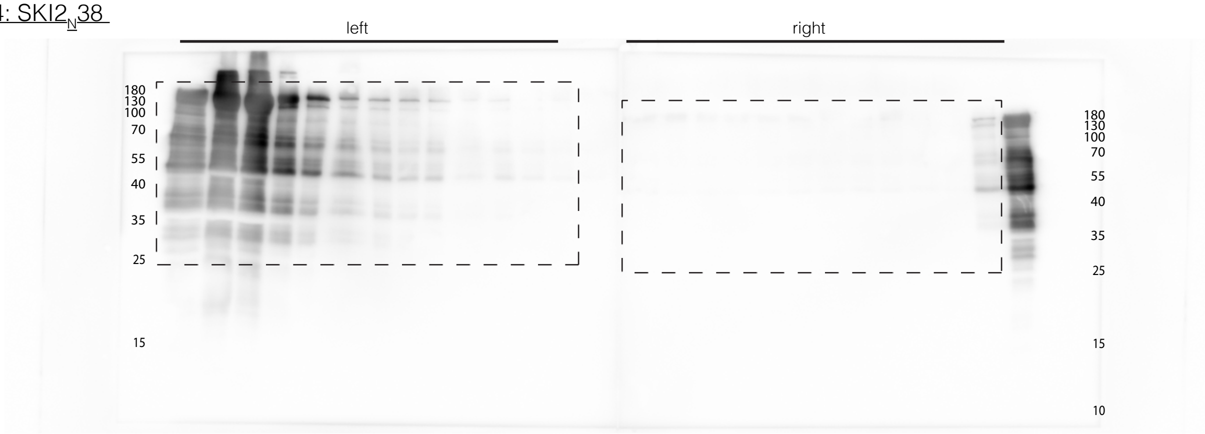

Supplement: Supplementary file 1 — Raw gels and western blot images. [file 41586_2024_8015_MOESM1_ESM.pdf]
