# Supplementary material for: Structural basis of mRNA decay by the human exosome–ribosome supercomplex
Source: Nature. 2024 Oct 9;635(8037):237–42. doi: 10.1038/s41586-024-08015-6 (PMC11540850; doi:10.1038/s41586-024-08015-6)
Supplement: Supplementary file 2 — Reporting Summary [file 41586_2024_8015_MOESM2_ESM.pdf]

nature portfolio

Corresponding author(s): Ingmar B. Schäfer & Elena Conti

Last updated by author(s): Jul 25, 2024

Reporting Summary

Nature Portfolio wishes to improve the reproducibility of the work that we publish. This form provides structure for consistency and transparency in reporting. For further information on Nature Portfolio policies, see our Editorial Policies and the Editorial Policy Checklist.

Statistics

For all statistical analyses, confirm that the following items are present in the figure legend, table legend, main text, or Methods section.

n/a	Confirmed	
☐	☒	The exact sample size (*n*) for each experimental group/condition, given as a discrete number and unit of measurement
☐	☒	A statement on whether measurements were taken from distinct samples or whether the same sample was measured repeatedly
☒	☐	The statistical test(s) used AND whether they are one- or two-sided<br>*Only common tests should be described solely by name; describe more complex techniques in the Methods section.*
☒	☐	A description of all covariates tested
☒	☐	A description of any assumptions or corrections, such as tests of normality and adjustment for multiple comparisons
☐	☒	A full description of the statistical parameters including central tendency (e.g. means) or other basic estimates (e.g. regression coefficient) AND variation (e.g. standard deviation) or associated estimates of uncertainty (e.g. confidence intervals)
☒	☐	For null hypothesis testing, the test statistic (e.g. *F*, *t*, *r*) with confidence intervals, effect sizes, degrees of freedom and *P* value noted<br>*Give P values as exact values whenever suitable.*
☒	☐	For Bayesian analysis, information on the choice of priors and Markov chain Monte Carlo settings
☒	☐	For hierarchical and complex designs, identification of the appropriate level for tests and full reporting of outcomes
☒	☐	Estimates of effect sizes (e.g. Cohen's *d*, Pearson's *r*), indicating how they were calculated

Our web collection on statistics for biologists contains articles on many of the points above.

Software and code

Policy information about availability of computer code

Data collection	SerialEM 4.0; Digital Micrograph 3.32; Focus 1.1.0
Data analysis	Motioncor2; GCTF 1.06; Gautomatch 0.56; RELION 3.1; ChimeraX 1.6.1; PHENIX 1.20; Coot 0.8.9; ImageJ

For manuscripts utilizing custom algorithms or software that are central to the research but not yet described in published literature, software must be made available to editors and reviewers. We strongly encourage code deposition in a community repository (e.g. GitHub). See the Nature Portfolio guidelines for submitting code & software for further information.

Data

Policy information about availability of data

All manuscripts must include a data availability statement. This statement should provide the following information, where applicable:

- Accession codes, unique identifiers, or web links for publicly available datasets
- A description of any restrictions on data availability
- For clinical datasets or third party data, please ensure that the statement adheres to our policy

Cryo-EM density maps that support the findings in this study have been deposited in the Electron Microscopy Data Bank (EMDB) and the Protein Data Bank (PDB) under the accession numbers: Map1/EXO10-SKI-80S (80S subtracted) EMDB: 51133, PDB: 9G8N; Map2/EXO10SKI-80S (60S subtracted) EMDB: 51139; Map3/EXO10-SKI-80S (full map) EMDB: 51132, PDB: (9G8M); Map4/SKI7-SKI238 EMDB: 51137, PDB: 9G8R; Map5/ EXO10-SKI-40S (EXO10-SKI2H + 40S subtracted) EMDB: 51136, PDB: 9G8Q; Map6/EXO10-SKI-40S (full map) EMDB-51134, PDB; 9G8O; Map7/EXO10-SKI-40S (40S + SKI gatekeeping module subtracted); EMDB 51135, PDB: 9G8;

Map8/Control EXO10-SKI-40S EMDB-51135. All other data are available within the main text or the Extended Data. Raw data and source images are available in the supplementary information.

Research involving human participants, their data, or biological material

Policy information about studies with human participants or human data. See also policy information about sex, gender (identity/presentation), and sexual orientation and race, ethnicity and racism.

Reporting on sex and gender	These data were not collected in this study
Reporting on race, ethnicity, or other socially relevant groupings	These data were not collected in this study.
Population characteristics	These data were not collected in this study.
Recruitment	Participants were not recruited for this study.
Ethics oversight	No approval number was required for this study.

Note that full information on the approval of the study protocol must also be provided in the manuscript.

Field-specific reporting

Please select the one below that is the best fit for your research. If you are not sure, read the appropriate sections before making your selection.

☒ Life sciences ☐ Behavioural & social sciences ☐ Ecological, evolutionary & environmental sciences

For a reference copy of the document with all sections, see nature.com/documents/nr-reporting-summary-flat.pdf

Life sciences study design

All studies must disclose on these points even when the disclosure is negative.

Sample size	No sample sizes were calculated. CryoEM data were collected over multiple days to yield a sufficient amount of particles and to obtain high resolution 3D reconstructions of the complexes of interest. The number of technical replicates in the extended data figure 1f was chosen based on the minimum amount required for statistical verification that the RNA to be used in structural studies was an appropriate substrate, although no calculations were performed.
Data exclusions	During cryoEM data processing, particles were excluded during 2D and 3D classification when missing clear secondary structure features in 2D class averages or 3D reconstructions.
Replication	Western blots, pulldown assays, and degradation assays were performed in independent triplicates to confirm similar results. Raw images and source data are shown in Supplementary.
Randomization	Randomization was not relevant to this study because we did not include human subjects or animals.
Blinding	Blinding was not relevant to this study because we did not include human subjects or animals.

Reporting for specific materials, systems and methods

We require information from authors about some types of materials, experimental systems and methods used in many studies. Here, indicate whether each material, system or method listed is relevant to your study. If you are not sure if a list item applies to your research, read the appropriate section before selecting a response.

Materials & experimental systems

n/a	Involved in the study	
☐	☒	Antibodies
☐	☒	Eukaryotic cell lines
☒	☐	Palaeontology and archaeology
☒	☐	Animals and other organisms
☒	☐	Clinical data
☒	☐	Dual use research of concern
☒	☐	Plants

Methods

n/a	Involved in the study	
☒	☐	ChIP-seq
☒	☐	Flow cytometry
☒	☐	MRI-based neuroimaging

Antibodies

Antibodies used	Monoclonal ANTI-FLAG clone M2 mouse antibody (Sigma-Aldrich, Cat. No. F3165); Polyclonal anti-mouse HRP-coupled antibody (Bio-Rad, Cat. No. 172-1011)
Validation	Monoclonal ANTI-FLAG antibody was validated against purified SKI-complexes, where the SKI3 subunit was FLAG tagged (Extended Data Fig. 9)

Eukaryotic cell lines

Policy information about cell lines and Sex and Gender in Research

Cell line source(s)	HEK293T - ATCC; Hi5 - Invitrogen (Thermo Fisher Scientific)
Authentication	None of the cell lines were authenticated.
Mycoplasma contamination	All cell lines tested negative for mycoplasma contamination based on regular testing.
Commonly misidentified lines (See ICLAC register)	No commonly misidentified cell lines were used in this study.

Plants

Seed stocks	No seed stocks were used in this study.
Novel plant genotypes	No novel plant genotypes were generated in this study.
Authentication	This is not applicable to this study.
